# A hybrid short read mapping accelerator

**DOI:** 10.1186/1471-2105-14-67

**Published:** 2013-02-26

**Authors:** Yupeng Chen, Bertil Schmidt, Douglas L Maskell

**Affiliations:** 1 School of Computer Engineering, Nanyang Technological University, Singapore, Singapore; 2 Institut für Informatik, Johannes Gutenberg University Mainz, Mainz, Germany

## Abstract

**Background:**

The rapid growth of short read datasets poses a new challenge to the short read mapping problem in terms of sensitivity and execution speed. Existing methods often use a restrictive error model for computing the alignments to improve speed, whereas more flexible error models are generally too slow for large-scale applications. A number of short read mapping software tools have been proposed. However, designs based on hardware are relatively rare. Field programmable gate arrays (FPGAs) have been successfully used in a number of specific application areas, such as the DSP and communications domains due to their outstanding parallel data processing capabilities, making them a competitive platform to solve problems that are “inherently parallel”.

**Results:**

We present a hybrid system for short read mapping utilizing both FPGA-based hardware and CPU-based software. The computation intensive alignment and the seed generation operations are mapped onto an FPGA. We present a computationally efficient, parallel block-wise alignment structure (Align Core) to approximate the conventional dynamic programming algorithm. The performance is compared to the multi-threaded CPU-based GASSST and BWA software implementations. For single-end alignment, our hybrid system achieves faster processing speed than GASSST (with a similar sensitivity) and BWA (with a higher sensitivity); for pair-end alignment, our design achieves a slightly worse sensitivity than that of BWA but has a higher processing speed.

**Conclusions:**

This paper shows that our hybrid system can effectively accelerate the mapping of short reads to a reference genome based on the seed-and-extend approach. The performance comparison to the GASSST and BWA software implementations under different conditions shows that our hybrid design achieves a high degree of sensitivity and requires less overall execution time with only modest FPGA resource utilization. Our hybrid system design also shows that the performance bottleneck for the short read mapping problem can be changed from the alignment stage to the seed generation stage, which provides an additional requirement for the future development of short read aligners.

## Background

High-throughput DNA sequencing technologies (such as Illumina sequencers [1]) have promoted the production of short reads with dramatically low unit cost. The explosive growth of short read datasets poses a challenge to the mapping quality and the execution speed. The main task of short read mapping is to align the reads to a given reference genome. However, mapping this large volume of data is a challenge for existing sequence alignment tools. The Smith-Waterman algorithm [2] is able to provide an accurate alignment result, but is too slow for the large volumes of data generated by current sequencers. Therefore, existing software tools often use a restrictive error model for computing the alignments to improve speed, whereas more flexible error models are generally too slow for large-scale applications. As the read length continues to increase and more errors are permitted in the final alignment, many current aligners are becoming less efficient. Our goal in this paper is to design, implement, and evaluate a new short read alignment (SRA) method with both high sensitivity and high throughput. To achieve these goals, our approach is based on implementing a sensitive alignment method following the “seed and extend” approach on an FPGA. Both high sensitivity (defined as the number of alignments found) and decreased computational processing time are significant advances to research in the area of Next-Generation-Sequencing (NGS).

“Seed and extend” is a frequently used heuristic in short read mapping implementations. The basic idea is simple: since only a limited number of errors are allowed for a significant alignment^a^ long exact match regions exist. Thus, discovering these exact matches (called common *k*-mers or seeds) before the alignment process can largely reduce the search space. Detection of these seeds is usually performed using two approaches: (i) indexing of the input read dataset and scanning through the reference genome, (ii) indexing of the reference genome and aligning each read independently.

There are several alignment tools based on the first approach (e.g. MAQ, ZOOM, SHRiMP). MAQ [3] performs an ungapped alignment that takes into account quality scores for each base. ZOOM [4] uses “spaced-seeds” in order to improve sensitivity. SHRiMP [5] combines the spaced seeds and the Smith-Waterman algorithm to align reads with even higher sensitivity. SOAP, WHAM, BFAST, and GASSST apply the second approach to conduct the alignment computation. SOAP [6] uses seeds (consecutive bases) and a hash lookup table algorithm to accelerate the alignment and is efficient to process alignments with a small number of gaps and mismatches. WHAM [7] uses a hash-based index method to quickly find potential hits and then applies bitwise operations to perform string matching. BFAST [8] uses multiple indices of the reference genome to increase sensitivity. GASSST [9] applies a series of filters of increasing complexity to quickly eliminate candidate hits with too many alignment errors. A drawback of these indexing methods is that the memory footprint is very large, particularly when the size of the reference genome or the reads approaches several billion. A third approach, based on the Burrows-Wheeler transform (BWT) [10], addresses this problem by applying an occurrence table and a suffix array to store the reference genome in a space-efficient way. Bowtie [11] employs a Burrows-Wheeler index which greatly reduces the memory consumption. Bowtie is one of the fastest alignment tools for short read alignment, but does not allow for indel (insertion and deletion) errors. BWA [12] is slightly slower than Bowtie, but allows indels in the alignment. SOAP2 [13] uses a bidirectional BWT to build the index of the reference genome and achieves a comparable alignment speed to that of Bowtie. The BWT-based methods use a backward search [14] to quickly locate exact matches. However, its search space increases dramatically if more errors are allowed. Therefore, this approach is generally efficient for low error rates.

As discussed above, short read mapping, particularly for very large datasets, is computationally challenging. Hybrid computing platforms offer the potential to improve algorithm performance, particularly algorithm runtime. Already, we have seen the development of hybrid short read alignment tools using the parallel computing capabilities of GPUs. SARUMAN [15] uses a NVIDIA graphics card to accelerate the time-consuming alignment step. SOAP3 [16] and CUSHAW [17] achieve performance improvements by parallelizing the BWT-approach on GPUs. [18] proposed a hybrid system combining both CPU and GPU to accelerate the phylogeny-aware alignment kernel.

FPGAs are also suitable candidate platforms for this application, due to their fine-grained pipelining and massive parallelism. However, short read mapping tools on FPGA are less common due to the design efforts required. Alachiotis *et al*. [19] proposed a FPGA-based short read alignment accelerator for the phylogenetic tree search, which is based on the PaPaRa [20] algorithm. Knodel *et al*. [21] developed a massively parallel structure to conduct the straightforward search for short reads in a reference database. However, gaps are not allowed in the design and as such it limits this design’s application scope. Fernandez et al. [22] designed a short read aligner based on BWT indexing. However, as it stores the table contents utilizing on-chip memory resources, the supported reference genome size is quite limited. Tang et al. [23] presented a heterogeneous short read aligner based on the algorithm used in PerM [24]. For its current version, the maximum working clock frequency is 175 MHz and gaps are not allowed. Olson et al. [25] designed another FPGA aligner based on the BFAST algorithm. The index data structure and candidate alignment locations (CAL) finder design improve the search efficiency. The final system achieves two orders of magnitude speedup against BFAST and an order of magnitude speedup against Bowtie with the help of 8 Virtex-6 FPGAs, ×16 PCIe buses, and DDR3 memory interfaces. Generally, it is very difficult to make a meaningful direct comparison between two different FPGA designs. The performance of an FPGA design is influenced by many factors, including the FPGA architecture and the original algorithm that was implemented. Unlike above designs, our FPGA aligner design is inspired by the concept of pre-filtering. In this paper, we present a hybrid short read aligner built on one Virtex5 FPGA chip with a similar structure to that of GASSST, but with faster processing speed.

## Methods

### Runtime profiling

Our hybrid aligner design follows the “seed and extend” strategy and indexes the reference genome. The “seed” stage identifies candidate regions with a high degree of similarity between the reference genome and each read sequence. The hash table lookup is a conventional method to quickly eliminate irrelevant regions. It can be easily implemented on the FPGAs due to its simple structure. The extension stage extends the seed in both directions. If indels are supported, the DP-based Needleman-Wunsch (NW) algorithm [26] can be applied. The NW algorithm provides a high degree of accuracy, but is also computationally expensive due to its quadratic search space. Based on our experience, the number of indels allowed is much less than that of substitutions for short read alignment. This provides us the opportunity to use another DP-based algorithm with a much smaller search space, the banded NW algorithm. To evaluate the performance bottleneck of the sequential NW-based short read mapping, we record the runtime of the three different stages, i.e. indexing, seed generation, and seed extension with the NW algorithm on a conventional CPU using a single thread. In this test, we use one million simulated reads of length 76 base-pairs (bps) each from the *E. Coli* genome and a 4% error rate. The results in Table 1 show that the extension stage is the most time-consuming part. Furthermore, the seed generation stage also occupies over 13% of the overall runtime. The extension stage is quite suitable for FPGA implementation, as it has a regular systolic architecture. The seed generation stage mainly consists of random memory accesses to a large lookup table. In our earlier version of the hybrid aligner design [27], we mapped the seed generation stage into software. The general purpose CPU pre-caches part of the data from the main memory to accelerate consecutive memory accesses. Unfortunately, the performance deteriorates significantly for random memory accesses. Our earlier experiments showed that this type of partitioning is unable to generate enough (*read*, *ref*) pairs for the extension stage on the FPGA. Thus, we have decided to construct a seed engine module to implement the seed generation operations on the FPGA to achieve better performance.

**Table 1:** Runtime performance for short read mapping with a single thread on an AMD 2.1 GHz CPU

	**Runtime (s)**	**Percentage of time spent**
Indexing	7.03	0.79%
Seed generation	120.14	13.5%
Extension	760.19	85.7%

### Parallel banded NW search

For two given letters *x* and *y* (bases or bps) over the nucleotide alphabet ∑ = {A, C, G, T}, we use the following scoring scheme: 0 for a match (i.e. if *x = y*), and 1 for a mismatch (i.e. if *x ≠ y*). Furthermore, the penalty for each indel error is also set to 1. The computation of the DP alignment matrix between a read sequence (*read*[1..*n*] ∈ ∑^*n*^) and a substring of the reference genome (*ref*[1..*m*] ∈ ∑^*m*^) is given in Equation (1) for 1 ≤ *i* ≤ *m* and 1 ≤ *i* ≤ *n*.

(1) $$S\left(i,j\right)=\min\{\begin{array}{c} S\left(i-1,j\right)\;+\;1\\ S\left(i-1,j-1\right)\;+\;\delta \\ S\left(i,j-1\right)\;+\;1 \end{array}$$

where *δ* = 0 if *ref (1) = read (j)*; otherwise, *δ* = 1. The DP matrix initializations are given by *S (i, 0) = i* and S (0, j) = j for 0 ≤ *i* ≤ *m* and 0 ≤ *j* ≤ *n*. The optimal global alignment score with respect to the given scoring scheme is then the value *S*(*m*,*n*). In fact, short read mapping can be considered as a semi-global alignment, where only the query read needs to be globally aligned. Thus, the optimal score is given by the minimal value in the last column of S, i.e. min{*S*(*i*,*n*)∣*i*∈{1,…,*m*}} and the gaps in the first column are omitted.

**Figure 1:**
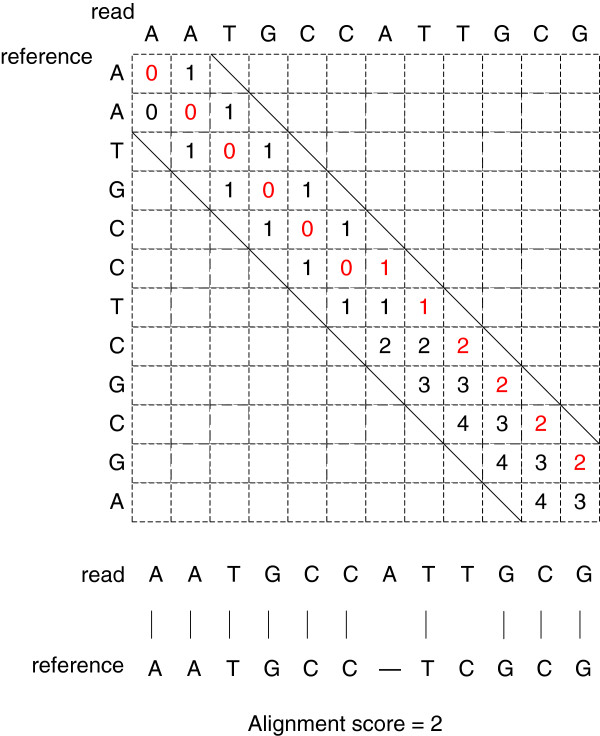
**The conventional banded semi-global alignment between two sequences “*AAGCCATTGCG*” and “*AATGCCTCGCGA*”.**

The search space for the conventional global alignment is of size *m × n*. The banded NW algorithm limits the search process around the main diagonal with a band width of *d*, which largely reduces the search space. However, the alignment score is still computed sequentially. Figure 1 shows a typical case for a banded semi-global alignment with a band width of one.

**Figure 2:**
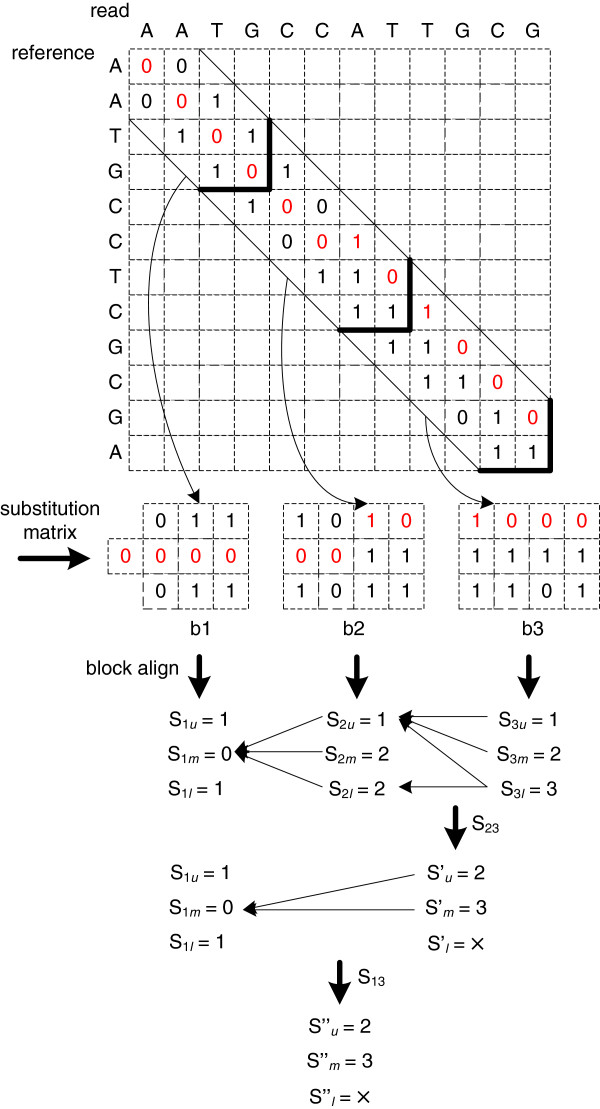
The parallel banded NW alignment.

To further utilize the parallelism inherent in FPGAs, we have modified the banded NW search to a fine-grained parallel version. Instead of computing the alignment score directly as in Equation (1), we first take only substitution errors between bases into account. The highlighted bases in Figure 2 indicate the best alignment between two sequences. As a good alignment permits only a limited number of errors, the optimal alignment score is usually related to the path with fewer 1’s, which gives an insight as to how to conduct the alignment in parallel. As the paired match information is independent of each other, we can divide the search space into multiple blocks and compute the alignment score for each block in parallel. As the best alignment in the block is not always part of the final alignment, we compute the alignment scores for three different diagonals at the same time with each block, labelled as (*S*_*u*_, *S*_*m*_, *S*_*l*_), to prevent possible sensitivity loss. We also record the start locations for each score labelled as {*u, m, l*} representing the upper diagonal, the main diagonal, and the lower diagonal. Afterwards, we concatenate the block scores to get the complete alignment score. Since the block scores are independent of each other, we use a tree structure to concatenate multiple block scores in parallel to reduce the computation time. Our parallel block alignment algorithm is similar to the Four-Russian speedup technique [28] for block alignment, but we employ a different block construction strategy. Instead of partitioning the search space into overlapping square blocks, we divide the search space into consecutive “v-shaped” regions, without any overlap. GASSST also introduces a tiled-NW filter to compute the alignment score in blocks. A small lookup table for 4 bp long sequence pairs is applied for the block score computation. The lower bound score is computed among three overlapping regions along the main diagonal. As the tiled-NW filter is the first stage of the cascading filters in GASSST, its performance for eliminating the candidate hits is quite limited. In contrast, our parallel block alignment algorithm can provide a high sensitivity on candidate elimination (see the experimental results). Figure 2 gives an example of our parallel banded NW search for two 12 bp sequences. The bold lines in Figure 2 construct three blocks for alignment computation. After block reformation, we get a substitution matrix for each block. The alignment score computation is determined by its substitution matrix. Assuming the threshold is three, if the alignment score is higher than the threshold, the alignment will be ignored and labelled with the symbol “×”. The block score computation is divided into two categories: the initial block alignment and the latter block alignment.

**Figure 3:**
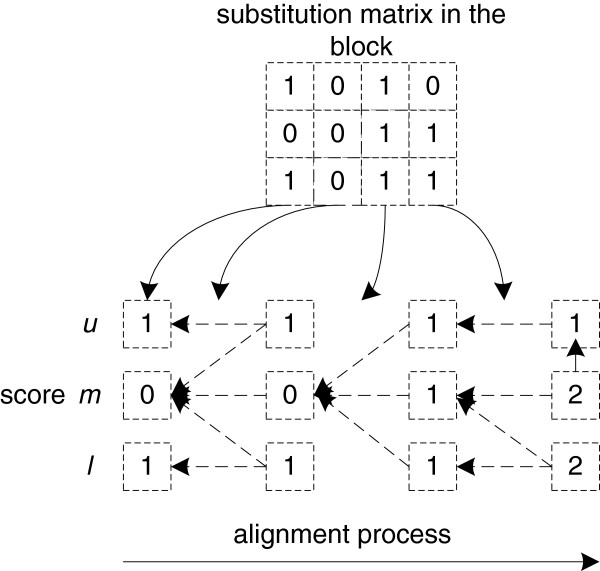
Alignment computation within the block.

The initial block score computation is the same as the conventional banded semi-global alignment. The block *b*_1_ score computation in Figure 2 belongs to this category. Its alignment score is *S*_1_ = (1, 0, 1) and the start location is (*m*, *m*, *m*). The score computation for block *b*_2_ and block *b*_3_ falls into the other category (later block alignment), and also follows Equation (1), but assumes that there are three possible start points (*u*, *m*, and *l*, respectively) for the alignment. Figure 3 shows the detailed computations steps for block *b*_2_. The dashed arrows in Figure 3 indicate the trace-back paths of the alignment. Based on these paths, we can determine the start location for each score in the last column of the score section. The final score for block *b*_2_ is *S*_2_ = (1, 2, 2) with a start location of (*m*, *m*, *m*). Following the same method, the alignment score for block *b*_3_ is *S*_3_ = (1, 2, 3) and the start location is (*u*, *u*, *ul*) (for the same alignment, there could be multiple start locations). In Figure 2, we use the arrows to represent the start locations after block alignment. For example, the arrow from S_3*l*_ to S_2*u*_ indicates the start location of ‘*u*’. By concatenating block *b*_2_ and *b*_3_, we get the alignment score *S*_23_ = (2, 3, ×) and the updated start location (*m*, *m*, *m*). Following the same strategy, we can get the score for all three blocks as: *S*_123_ = (2, 3, ×). Then, the final alignment score is 2 (the same as the result of the conventional banded NW algorithm shown in Figure 1). For the conventional algorithm, it requires at least 12 cycles to get the alignment score. In contrast, our method only requires 6 cycles (4 cycles for block alignment and 2 cycles for concatenation). Thus, as the read length increases, we can expect greater computational efficiency. In practice, we have designed an FPGA aligner including twelve blocks, where each block can conduct an 8 bp alignment.

### Hybrid system architecture

**Figure 4:**
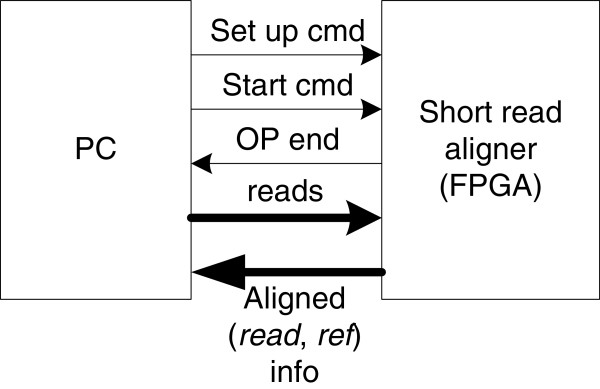
The hybrid system architecture.

The overall hybrid system structure is shown in Figure 4. In our design, we use a constant-length seed model and index the reference genome. Thus, the reference genome only needs to be processed once and the results can be reused for different read datasets. Therefore, indexing is treated as a pre-processing stage, which is not included in the overall alignment work flow.

The complete system is running on a DRC coprocessor system [29] and the host PC communicates with the FPGA chip through the HyperTransport interface. The main tasks for the host PC are simple and are not compute-intensive, consisting of: read coding (i.e. converting nucleotide bases into a binary format, two bits for each base) and transferring the coded reads to the short read aligner; sending operating commands to the aligner; receiving the alignment results and writing them to an output file. The short read aligner (available at http://code.google.com/p/fpga-sra-core/downloads/list) conducts the seed generation and extension operations on the FPGA chip.

The hybrid system includes two working states, the initialization state and the processing state. In the initialization state, the host PC first configures the aligner’s parameters through the use of setup commands. The FPGA aligner design supports different read lengths, which is controlled by the *length_setup* command. Although currently the maximum read length is 100 bps, it is relatively simple to modify the aligner to support larger lengths. The threshold score is set based on the *thresh_setup* command before the alignment start. Unlike other designs which conduct the alignment read by read, our hybrid system processes the alignment chunk by chunk. In our implementation, the host PC processes 1,000 reads per iteration. The coded read chunk is stored in FPGA BRAM resources. For 1,000 reads of length 100 bps, this consumes 400kbit of memory space (including the forward sequence and reverse complement sequence). The memory consumption is thus moderate and will not compete for resources required for subsequent stages. When the read chunk is ready, the host PC sends a *start* command to start the FPGA aligner. When chunk processing is finished, the FPGA aligner sends back an *operation_done* flag to inform the host PC. Based on our observation, the processing speed of the host PC is slower than that of FPGA aligner. Thus, to apply multi-threading on the read coding part can further improve the overall performance. An experiment related to multi-threading is given in “Results and Discussion” section.

**Figure 5:**
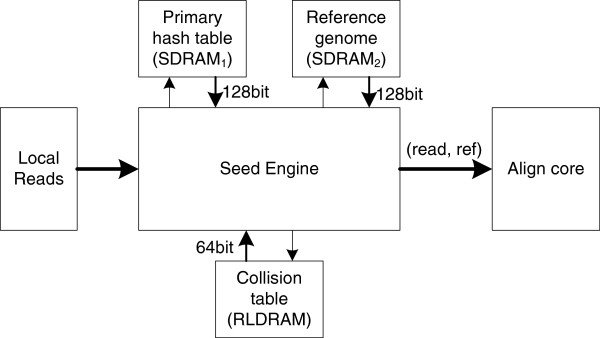
The short read aligner structure.

Our short read aligner is composed of two major FPGA components: the Seed Engine and the Align Core. The Seed Engine identifies all (*read*, *ref*) candidates with high similarity. The Align Core implements our parallel banded NW algorithm. The overall structure of the short read aligner is shown in Figure 5. Each of the off-chip DDR2 SDRAM memories provides 2Gbytes of storage with a bandwidth of 128bits, while the RLDRAM memory provides 256Mbytes storage with a bandwidth of 64bits. Two tables are attached to the Seed Engine. Both of them are generated by the indexing of the reference genome. The reference genome itself is also stored in the off-chip memory to generate (*read*, *ref*) pairs. The *Local Reads* module stores the coded read chunk data using on-chip memory.

### Seed engine

The hash table construction is the main task for the Seed Engine design. Software tools (e.g. GASSST) usually apply a flexible seed model, i.e. for different read lengths using different seed sizes. The advantage of the flexible seed model is that it can avoid generating too many candidate regions and moderate the work burden for the extension stage. However, the flexible seed model also requires repetitive indexing of the reference genome.

In our Seed Engine design, the constraint on the number of candidate regions is less stringent, since the Align Core can provide very fast alignment computation. Thus, a much simpler constant seed model is used. To evaluate the influence of different seed models, we record the number of duplicate seeds and alignments with different seed length settings in Table 2 (using the GASSST software implementations with one million simulated 76 bp reads against the *E. Coli* genome).

**Table 2:** The number of duplications and the identified alignments for different seed lengths using GASSST

**Seed length**	**# of duplications**	**# of alignments**
12 bp	1,260,817	1,076,671
13 bp	504,104	1,049,721
14 bp	217,461	1,029,516
15 bp	124,197	1,028,288

Table 2 shows that a longer seed length can effectively reduce the number of duplications, but the alignment sensitivity drops correspondingly. As duplicate seeds require extra computation time for hash table access, we arbitrarily choose 15 bp as the seed length to avoid a large number of duplications while slightly sacrificing the alignment sensitivity. To quickly identify candidate regions, two requirements exist for the hash function selection: (i) a simple hash function representation, and (ii) a reduced number of collisions. A complex hash function would compete with the hardware resources needed for the later extension stage implementation. Furthermore, if there are too many collisions, the additional off-chip memory accesses will reduce the overall performance.

**Figure 6:**
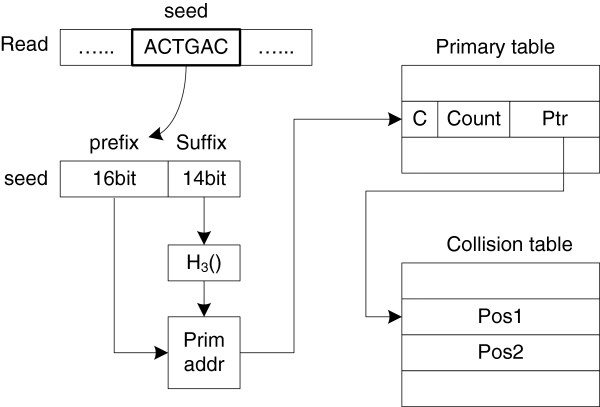
The hash query data path.

We apply the bucket hash strategy with hash functions chosen from the *H*_3_ family [30]. The bucket hash first divides the seeds into multiple buckets. A separate hash function is applied for individual buckets to guarantee that there are only a limited number of hash collisions. A seed is divided into two parts: the prefix and the suffix. Figure 6 gives an example of the hash query data path for a 15 bp seed. As the DNA alphabet size is only four characters, 30bits is enough to represent a 15 bp seed. The first 16bits are used as the prefix to construct the buckets; the remaining 14bits (suffix) are used for hash computation. The hash table address is computed by combining both the prefix and the hash value. As collisions only appear among seeds in the same bucket, the bucket hash strategy can effectively reduce the number of collisions.

To avoid sensitivity loss for seeds which collide, an additional table, called the collision table, is also constructed. The definitions of the primary table and the collision table fields are:**C** 1bit, the collision flag**Count** 7bits, the total number of seeds that were hashed to this slot**Ptr** 24bits, if the collision flag is ‘0’, it indicates the position in the reference genome otherwise, it indicates the start location in the collision table**Pos **24bits, the match position in the reference genome

For the *E. Coli* reference genome, the primary table consumes 64 K × 4 K × 32bits of memory and the collision table consumes 421623 × 32bits of memory. As both the primary hash table and the collision table are too large for the on-chip memory, the primary hash table is stored in DDR2 SDRAM_1_ and the collision table is stored in RLDRAM. Storing them in separate memories allows us to pipeline the hash query rather than waiting for a complete query operation. Once a match position in the reference genome is found, the Seed Engine will extract the related base information from SDRAM_2_ (the reference genome) to generate the valid (*read*, *ref*) pair for the extension stage.

As the Seed Engine computation refers to data transfer between the FPGA and off-chip memories, its performance is largely influenced by these I/O operations. The estimated highest clock frequency for the Seed Engine is around 250 MHz (reported by the Xilinx synthesis tool).

### Align core

**Figure 7:**
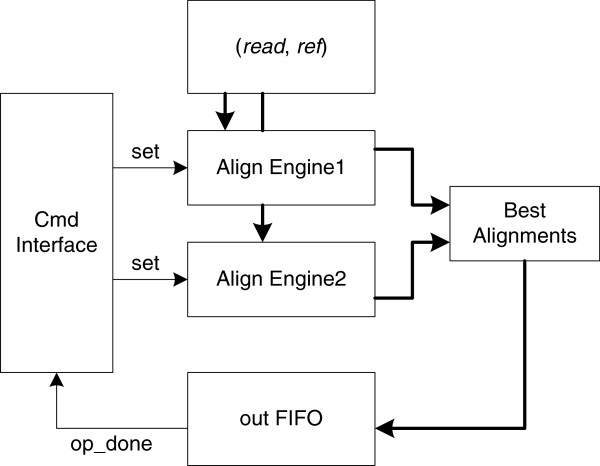
**The *Align Core *inner structure.**

The inner structure of the *Align Core* module is shown in Figure 7. It consists of two alignment engines, a command interface, a best alignments module, and an output FIFO. The command interface sets the operation parameters of the align engine and monitors the *operation_end* flag. The output FIFO temporarily stores the match (*read*, *ref*) information. The Alignment Engine performs the alignment computation.

**Figure 8:**
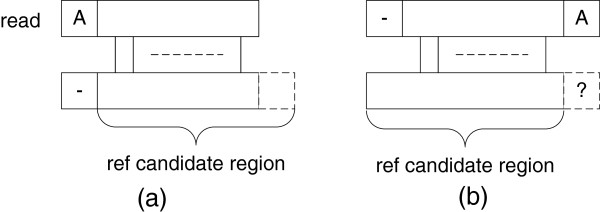
Special cases for semi-global alignment.

Normally, a single alignment engine is enough for the banded NW alignment, but in some occasional situations (e.g. characters outside the reference candidate region), the alignment score will be incorrect. Figure 8 gives an example of such special cases.

In Figure 8, case (a) is missing the extra character to the left of the candidate region. In case (a), the final score will be *S* + 1 (*S* indicates the candidate region alignment score, 1 is the initial insertion) for the normal search. However, if the character to the left of the candidate region happens to be ‘A’, the correct alignment score will change to *S*. This will introduce a false negative answer in the final results. To prevent possible sensitivity loss, we expand the search space by applying two alignment engines. One is for the normal search; the other searches the reference region shifted one base to the left. The “best alignments” module chooses the best alignment score between these two alignment engines and at the same time removes the duplicate results. In contrast, case (b) is missing extra characters to the right of the candidate region. As fewer nucleotides are computed, the alignment score could be less than its actual value, which adds false positive answers to the final results. To solve this problem, we also load one extra base to the right of the candidate region and compare it with the last character of the read. The alignment score from the lower diagonal will be updated with the extra base compare result.

**Figure 9:**
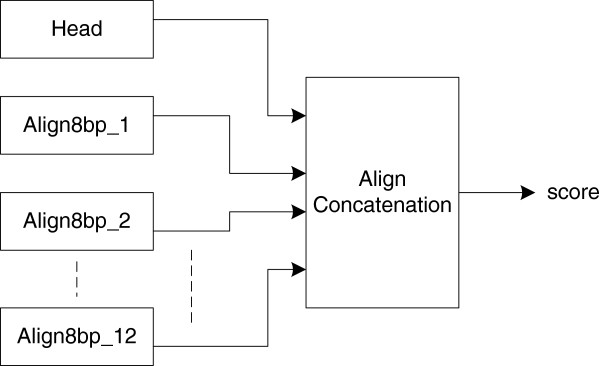
The alignment engine architecture.

The alignment engine design (shown in Figure 9) follows the parallel banded NW algorithm mentioned in Section II. It consists of three components: a head processing module, twelve smaller alignment modules (*Align8bp*), and a concatenate module.

**Figure 10:**
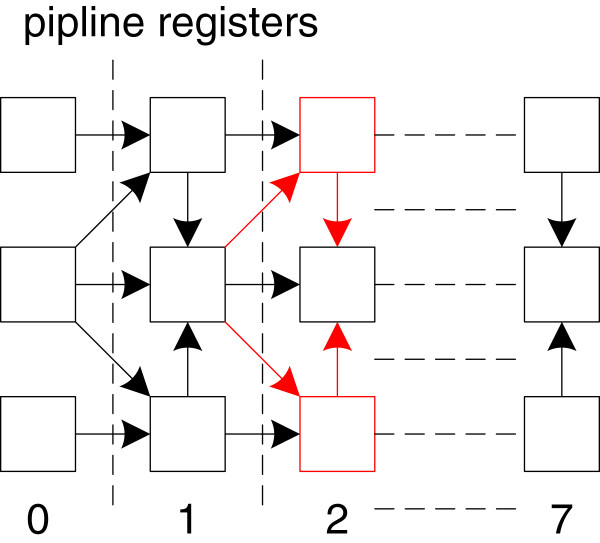
The Align8bp module inner structure.

The head-processing module conducts the semi-global alignment for the first four bases. Twelve *Align8bp* modules can support up to 96 bp alignment computation. The *Align8bp* module conducts the alignment computation using a substitution matrix of size 3 × 8 for each block. Its inner structure is shown in Figure 10. Each cell in Figure 10 is initialized with the value in the substitution matrix. The alignment score is updated along the path labeled in the inner structure. Meanwhile, each cell also records the updated start location based on the score computation result. When all computations are done, the last column will report the block alignment scores and corresponding start locations.

**Figure 11:**
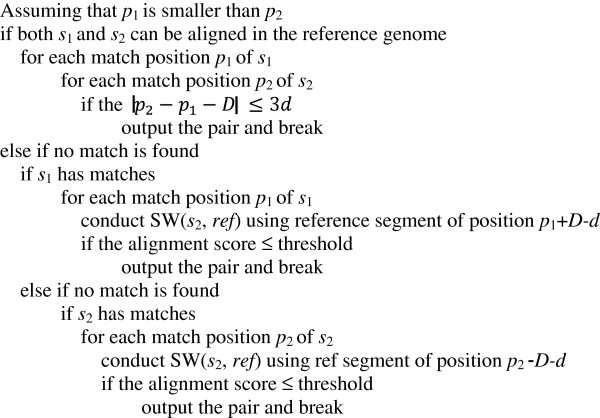
Pseudo code for the pair-end alignment.

The most time consuming path for the *Align8bp* module is updating the main diagonal score. We have labelled the critical path with red arrows in Figure 10. To further improve the *Align8bp* module’s performance, we insert extra registers to shorten the critical path. The alignment concatenation module design follows the method shown in Figure 2 and it also uses a tree structure to reduce the computation latency.

To support reads longer than 100 bp, we can simply duplicate the *Align8bp* module to extend our FPGA aligner’s processing capability. For alignments allowing more gaps, the parallel block algorithm still works except that the search space needs to be expanded. Our current FPGA aligner only supports alignments with a band width of one. To cope with more gaps, we need to expand our search space by adding more cells into the *Align8bp* module (e.g. if two gaps are allowed, the *Align8bp* module will require 5 × 8 cells in total; the connection between each cell is still similar to the one shown in Figure 10).

### Paired-end alignment

Unlike single-end short read alignment, the paired-end alignment problem involves a pair of short read datasets and two parameters, an outer distance *D* and a standard variation *d*. Similar to the single-end alignment, each read in the pair (*s*_1_, *s*_2_) will firstly be aligned individually, which can be accomplished utilizing our FPGA-based short read aligner. Define that *p*_1_ is the start location in the reference genome that read *s*_1_ is aligned; *p*_2_ is the start location in the reference genome that read *s*_2_ is aligned. Then, an additional pairing scheme is applied to complete the paired-end search on the host PC. The pairing scheme includes three cases: (1) both *s*_1_ and *s*_2_ have matches in the reference genome; (2) only *s*_1_ has matches in the reference genome; (3) only *s*_2_ has matches in the reference genome. The pseudo code for the pairing scheme is shown in Figure 11. For each SW(·) operation in the pseudo-code, we conduct the forward search and the reverse complement Smith-Waterman alignment of the short read to increase sensitivity.

## Results and discussion

We have implemented the FPGA aligner using Verilog HDL and have targeted it to a Xilinx Virtex5 LX330 device. The design consumes 71,744 slice registers (34% of the available register resources), 79,552 slice LUTs (38% of the available LUT resources), and 133 36 kb BRAM blocks (46% of the available on-chip memory space). The FPGA aligner works at 200 MHz, which maximizes the I/O data transfer. Several experiments are conducted to evaluate the FPGA aligner’s performance at this clock frequency setting. The performance of our FPGA aligner is compared with GASSST (version 1.26) and BWA (version 0.5.9). The fundamental work flow of our FPGA aligner design is similar to that of GASSST, except that GASSST uses multiple pre-filters to eliminate irrelevant data before the computation intensive global alignment. BWA uses a totally different strategy (a BWT-based backward search) to conduct the short read alignment computation. It is probably one of the fastest aligners to date for alignments with a low error rate and is also a popular choice in the bioinformatics community. Thus, we also include BWA in the performance evaluation. Gaps are allowed in all three aligners. All tests are performed on a quad-core (each core running at 2.1 GHz) AMD Opteron processor with 8Gbyte RAM running the Linux OS. The reference genome is the *E. Coli* NC_008253 dataset with 4.9 million residues. We have generated several simulated short read datasets using the *wgsim* utility program in the *SAMtools* package [31] (version 0.1.17) with different error rate settings. In all experiments, the identification percentage is set to 90% and the number of gaps allowed is one. For example, for a 36 bp read alignment, at most three errors are allowed (at most one indel error and the rest are substitutions). The other parameters for GASSST and BWA are set to the default values.

To analyze the performance bottleneck for our hybrid system, we first measure the cumulative execution time of different parts of our system running with a single CPU thread. The results are shown in Table 3 using a 76 bp single-end read dataset with 4% error rate. The results show that the host PC computation occupies 69% of the overall execution time, which is related to read coding and match output. This indicates that the read coding and the match output is now the bottleneck for our hybrid system design. Further improvements on the FPGA aligner cannot improve the hybrid system’s performance significantly using just a single thread. However, the system performance can be improved using multi-threading.

**Table 3:** Hybrid system runtime profile

	**Runtime**	**Percentage of time spent**
Host PC	22.22 s	69%
FPGA aligner	9.78 s	31%

Additional experiments are also prepared to give a more thorough analysis of the FPGA aligner design. The second experiment is to evaluate the Align Core’s processing capability. We compare the runtime performance between the filtration part in GASSST and our Align Core, assuming that the input data for each of them is available. As this experiment only compares the “alignment” section runtime, the runtime performance of BWA is not included. Three small short read datasets (36 bp) of varying error rates are used. Each dataset contains one million reads. Table 4 reports the runtime comparison under different conditions. The GASSST filter runtime includes all filtration stages from the tiled-NW filter to the real NW filter computation using a single thread. The Align Core runtime also includes data transfer from off-chip memory to FPGA.

**Table 4:** Alignment runtime comparison for one million reads

	**4% error rate**	**6% error rate**	**8% error rate**
GASSST	55 s	46 s	37 s
Align Core	1.6 s	1.47 s	1.31 s
Speedup	34	31.3	28

Table 4 shows that the FPGA Align Core provides a much better runtime performance for the alignment computation (around 30 times faster). As the filtration (extension) stage is accelerated by the Align Core, it is no longer the performance bottleneck for the “seed-and-extend” strategy. However, the performance of the Align Core alone does not represent the complete performance of our hybrid system. To make a fair evaluation on the hybrid system’s performance, we have compared the complete execution time of GASSST, BWA, and our hybrid aligner using one million reads with 4% error rate with different read lengths (36 bp, 76 bp and 100 bp). We also record the performance under multi-threaded conditions. The results are shown in Table 5.

**Table 5:** Execution time comparison among different thread conditions

	**36 bp read data set**			**76 bp read data set**			**100 bp read data set**		
Threads	1	2	4	1	2	4	1	2	4
Hybrid aligner	16 s	11 s	11 s	30s	21 s	21 s	38 s	28 s	28 s
GASSST	110 s	58 s	42 s	216 s	116 s	78 s	268 s	145 s	96 s
BWA	64 s	34 s	22 s	150 s	88 s	54 s	214 s	104 s	71 s
Speedup_1_	6.8	5.3	3.8	7.2	5.5	3.7	7.1	5.2	3.4
Speedup_2_	4	3.1	2.0	5.0	4.2	2.6	5.7	3.7	2.5

(Note: speedup_1_ indicates the runtime comparison between the hybrid system and GASSST; speedup_2_ indicates the execution time comparison between the hybrid system and BWA).

The performance of our hybrid aligner reported in Table 5 is partially multi-threaded, i.e. different threads share the same FPGA aligner which only processes data from one thread at a time. Once a thread offloads the operations to the FPGA aligner, this thread needs to wait for the FPGA’s “computation done” flag. At the same time, other threads can keep encoding the short reads. As the FPGA aligner’s computation time is much shorter than that of read encoding, the waiting time for the thread is acceptable. As the BWT index can be reused for the same reference genome, the execution time of BWA in all our tests only includes *aln* and *samse* (*sampe* for paired-end alignment). BWA provides a faster processing speed than GASSST by taking advantage of the BWT-based search. Our hybrid aligner achieves a speedup of 7 over GASSST and of 5 over BWA with a single thread. The execution time speedup is less than that reported by the *Align Core* alone. This indicates that the Align Core is not fully utilized. When alignment computation is no longer the performance bottleneck, two other factors are responsible for the system’s overall performance: the read coding and the Seed Engine processing. The read coding computation is simple, but the total number of bases is large. Therefore, we utilize Streaming SIMD Extensions 2 (SSE2) instructions to minimize its computation time. Notice that there is no performance improvement for our FPGA aligner, when the number of threads changes from two to four. To find out the performance bottleneck for the current design, we further record the runtime consumed by the FPGA aligner under different thread conditions. Table 6 shows the runtime performance for the 100 bp case.

**Table 6:** The runtime composition for 1 million 100 bp reads

	**1 thread**	**2 threads**	**4 threads**
Total execution time	38 sec	28 sec	28 sec
FPGA aligner runtime	13.4 sec	21.65 sec	21.57 sec
Proportion	35.2%	77%	77%

Table 6 results show that when two threads are applied, the FPGA aligner’s runtime is almost doubled and its proportion jumps from 35.2% directly to 77%. This indicates that if more threads are applied, the FPGA aligner will be the performance bottleneck, which is proven by the zero performance improvement of the 4 thread case. As the *Align Core* is no longer the performance bottleneck for the FPGA aligner, the *Seed Engine* is the only explanation for such a performance limitation. The seed engine computation requires frequent off-chip memory accesses (multiple cycles are required for a complete off-chip memory access), which intrinsically limits its runtime performance. Since the processing speed is limited by the off-chip hash table access, several methods exist to further improve the design’s performance: (i) using FPGA platforms with multiple off-chip memory interfaces (thus allowing more hash lookups at the same clock cycle), or (ii) using FPGA chips with larger on-chip memories (the collision table can then be stored using BRAM; thus a single off-chip access will be enough for a hash query). As the resource consumption of our FPGA aligner design is less than 50% of the total FPGA resources, it is possible to duplicate the *Align Core* module or even the full FPGA aligner module on the same FPGA chip. However, the limited number of off-chip memory interfaces in the current experimental platform cannot generate any more (*read*, *ref*) pairs in the *Seed Engine* module. An FPGA board with more interfaces would eliminate this bottleneck, bringing additional performance improvements.

Runtime testing with a single error rate might not be enough to explore the performance of the three programs. Thus, we also test the runtime performance under different error rate conditions using simulated read datasets with a single thread. The performance is shown in Table 7.

**Table 7:** Runtime performance under different error rate conditions

	**76 bp**			**100 bp**		
Error	2%	4%	6%	2%	4%	6%
Hybrid aligner	30s	30s	29 s	37 s	38 s	37 s
GASSST	227 s	216 s	199 s	292 s	268 s	253 s
BWA	109 s	150 s	162 s	153 s	214 s	221 s
Speedup_1_	7.6	7.2	6.8	7.9	7.1	6.8
Speedup_2_	3.6	5.0	5.6	4.1	5.7	5.9

From Table 7, we can find that more execution time is consumed for all three alignment tools, as a longer read length is applied. This is because a longer read length leads to a larger search space, which naturally requires more computation time. Another interesting observation is that, under different error rate conditions, our hybrid aligner provides a relatively stable performance. In contrast, GASSST shows performance improvements and BWA shows performance deterioration, as the error rate increases. The search strategy used within the tools can explain such a difference. BWA applies the BWT-based search algorithm, which consumes additional computation time when an error occurs. Therefore, its computation time will increase, if more errors exist in the read dataset. The GASSST computation is based on the “seed and extension” strategy. As the error rate increases, the number of seeds in the read decreases, which reduces the computation time required in the extension stage. Our hybrid system also uses the “seed and extension” strategy, but the FPGA aligner computation only occupies a small portion of the total runtime (see Table 3). Thus, the error rate change only has limited influence on the execution time.

Besides investigating the speed of the FPGA aligner, we also examine the alignment quality among the different tools based on two factors: (i) the total number of alignments found (sensitivity) and, (ii) the number of true alignments found (accuracy). Since we know the exact positions for each read in the simulated datasets, the correctness of the reported alignments can be easily evaluated by comparing the position information. In practice, if an alignment position is within a maximal distance *d* (*d* = 5 in our evaluation) to the correct position, this alignment will be treated as a correct alignment. Table 8 shows the quality comparison using one million reads with different error rate settings.

**Table 8:** Alignments results for different read lengths and error rate conditions

		**BWA**			**GASSST**			**FPGA**		
		**2% err**	**4% err**	**6% err**	**2% err**	**4% err**	**6% err**	**2% err**	**4% err**	**6% err**
76 bp	# of aln	964,053	831,634	643,670	990,842	966,001	863,848	993,057	971,367	873,804
	true aln	950,293	819,522	634,386	976,482	951,587	851,162	977,722	956,785	861,025
100 bp	# of aln	964,005	835,099	655,336	990,970	976,574	905,030	993,701	988,114	944,693
	true aln	951,425	823,850	646,444	977,781	963,265	892,342	979,536	974,243	931,325

The alignment results show that BWA’s sensitivity drops dramatically compared to GASSST and our hybrid aligner, when the bases error rate increases. In contrast, both GASSST and our hybrid aligner maintain a relative stable performance: over 96% sensitivity for a 4% error rate and over 85% sensitivity for a 6% error rate. All three tools provide over 98% alignment accuracy. The alignment quality of our hybrid aligner is better than that of GASSST under the above test conditions, particularly at the higher error rate. The reason for the improvement is related to the seed model. GASSST uses a flexible seed model to ease the alignment computation. For longer read lengths, GASSST will use longer seed lengths (e.g. a 16 bp seed for the 76 bp read dataset; and a 19 bp seed for the100 bp read dataset). In contrast, our FPGA aligner uses the constant seed model (a 15 bp seed length), which can provide extra sensitivity for longer read datasets.

We have also conducted a further performance comparison of GASSST, BWA, and our hybrid system using larger read datasets. The simulated read datasets are 20 million sequences of length 76 bp and 100 bp, with a 4% error rate. The alignment performance is shown in Table 9 for a single thread implementation.

**Table 9:** Alignment performance for 20 million read datasets with 4% error rate

		**76 bp**			**100 bp**	
	**# of aln**	**true aln**	**exe time**	**# of aln**	**true aln**	**exe time**
Hybrid aligner	19,424,609	19,137,326	9 min 54 sec	19,760,231	19,484,102	12 min 32 sec
GASSST	19,318,463	19,034,347	67 min 14 sec	19,524,380	19,255,520	86 min 16 sec
BWA	16,627,675	16,387,736	50 min 48 sec	16,687,423	16,464,717	70 min 57 sec

The experimental results show that BWA only achieves around 83% sensitivity, while GASSST and our hybrid system achieve around 97% sensitivity. This indicates that both GASSST and our hybrid system are more effective in finding good alignments under this error setting. On the runtime performance side, BWA provides a faster processing speed than GASSST by taking advantage of the BWT-based search. Our hybrid system achieves the fastest processing speed, while maintaining the sensitivity at a high level. In addition to the simulated short read datasets, we have also evaluated the alignment performance using a real dataset (the short read sample from SRR519953, where the N’s are replaced with a random character, giving a total of 1,884,895 paired reads). In this case, a separate program is used to perform the substitution. The original read length within SRR519953 is 101 bp. As our design only supports a maximum 100 bp short reads, we chop one character from the 3^′^ end. For the single-end alignment, we use the first 1,884,895 short reads to conduct the test. Table 10 records the alignment quality and the runtime performance with a single thread for the three aligners. It is clear that our hybrid aligner reports more alignments than the other two aligners with a faster processing speed (over 6 times speedup).

**Table 10:** Single-end short read alignments using real dataset SRR519953

	**# of alignments**	**Aligned rate**	**Execution time**
Hybrid aligner	1,522,697	80.7%	65 sec
GASSST	1,477,911	78.4%	467 sec
BWA	1,472,773	78.1%	444 sec

As GASSST does not support paired-end alignment, the performance comparison is made only between BWA and our hybrid aligner using one million simulated short reads. Before the performance analysis, we further introduce several parameters to evaluate the alignment quality: precision (defined as the number of true alignments/ total alignments), recall (the number of true alignments/ the number of reads), and F-score ( $\mathrm{F-score}\;=\;\frac{2\;\times \;\mathrm{precision}\;\times \;\mathrm{recall}}{\mathrm{precision}\;+\;\mathrm{recall}}$). The F-score combines the influence of precision and recall together to evaluate the alignment quality. The comparison results are shown in Table 11. The paired-end read dataset is generated using the *wgsim* utilities with an outer distance of 200, a standard variation of 20, and a 4% error rate. The performance is tested with a single thread. Table 12 records the performance achieved using the real SRR519953 dataset.

**Table 11:** Paired-end alignment performance

**100 bp simulated reads**					
	**Aligned**	**Precision**	**Recall**	**F-score**	**Execution time**
BWA	1,940,132	99%	96.03%	97.49%	548 sec
Hybrid aligner	1,962,404	97.9%	96.05%	96.97%	108 sec

**Table 12:** Paired-end alignment with real dataset

	**# of alignments**	**Aligned rate**	**Execution time**
BWA	3,002,675	79.6%	1037 sec
Hybrid aligner	2,841,262	75.3%	300 sec

Table 11 results show that our hybrid aligner finds more paired-end alignments than BWA, but with a slightly worse precision performance for one million simulated 100 bp reads. We notice that the speedup achieved by pair-end alignment is smaller than that of single-end alignment. This is related to the pairing strategy applied by BWA which further utilizes the advantage of the BWT index to accelerate the pairing process. In Table 11, our hybrid aligner reports more alignments, but BWA reports more alignments in Table 12. We believe such difference also routes on the pairing strategy. The reported alignments can be increased simply by increasing the search space (O(*l*_1_*l*_2_), *l*_1_ is the read length; *l*_2_ is the candidate region) in the pairing process, but the execution time will also increase correspondingly.

The above experiments show that our hybrid system achieves a significant performance improvement when testing with a small reference genome (the E. Coli reference genome). While our hybrid system design is independent of the reference genome, the restricted memory size of the experiment platform (and not the implementation) limits the possibility to test with a larger reference genome, such as the human genome (with over three billion bases). If no partitioning is applied for the human genome and we use the minimal perfect hash function to construct the hash table, the system requires at least 12GByte memory for the hash table (three billion entries for the table with each entry pointing to a 32bit table content). In contrast, our experimental platform only has 2GByte of off-chip memory for the hash table, which is not large enough to support the human genome. In practice, the memory footprint is likely to even larger than 12GByte, as it is difficult to get a minimal perfect hash function for the human genome. A different FPGA platform with more off-chip memory could support the short read mapping against the human genome using our proposed architecture. Another possible solution to support human genome is to apply BWT-based methods in the seed generation stage design to reduce the memory consumption.

A number of short read aligners built on other hybrid platforms, such as GPUs, have appeared in the research literature. We have further compared our FPGA-based aligner to the GPU-based aligner CUSHAW [17] (version 1.0.40) (using default parameters) on an NVIDIA Tesla C2075 GPU attached to an Intel Xeon quad-core CPU 3.33 GHz with 8 GB RAM running the 64bit Ubuntu 12.10 OS. Similar to the previous experiments, the short read dataset consists of one million 76 bp short reads with 4% base error rate. The reference genome is the *E. Coli* genome. Table 13 shows the runtime performance for the two aligners running with a single CPU thread as well as some key features of the two experiment platforms.

**Table 13:** Performance comparison between a GPU-based aligner and our hybrid aligner

	**CUSHAW**	**hybrid aligner**
Runtime performance	15 sec	30 sec
Clock frequency*	1.15 GHz	200 MHz
Attached memory	6 GB	4 GB
Memory I/O	384-bit GDDR5	128-bit DDR2

* The clock frequency is the clock for the Tesla C2075 board and the FPGA chip, respectively. The GPU specifications are from [32].

In this experiment, CUSHAW is twice as fast as our hybrid aligner. This runtime speedup is achieved by a combination of both the mapping algorithm within CUSHAW and the GPU platform. These advantages include: (1) BWT-based indexing, which largely reduces the memory footprint; (2) disallowing gaps, which reduces the search space; (3) a wider memory I/O, as short read mapping requires intensive memory accesses, this will definitely be an advantage; (4) the much higher clock frequency of the GPU provides a better runtime performance. Additionally, the NVIDIA Tesla C2075 board was released in 2011. In contrast, the LX330 FPGA was released in 2006, which means it is at least two generations older than the C2075. For example, when mapping our FPGA aligner to the up-to-date Xilinx Virtex-7 XC7VX1140T chip, the Xilinx tool chain estimated a working frequency of 264 MHz (1.3 times higher than our current working frequency). In addition, this chip contains 68Mbit of BRAM (it is large enough to store the collision table and the *E. Coli* genome using on-chip memory). Therefore, we can use SDRAM_2_ (as shown in Figure 5) to store another copy of the primary hash table to further double the (*read*, *ref*) pair generation rate. Furthermore, when we replace the DDR2 memory with the DDR3 memory on the Virtex-7, the memory I/O bus clock increases from 200 MHz to 400 MHz resulting in an additional 2 times speedup. Thus, we can expect approximately 5.2 times performance improvement in total (1.3× from FPGA working frequency, 2× from duplicated primary hash table, 2× from faster DDR3 memory interface).

Both FPGAs and GPUs are powerful platforms for high performance computing, but they have advantages for different types of applications. The advantage of GPUs lies in the many hundreds of cores, the different types of memories and the fast data transfer interfaces; whereas the advantage of FPGAs lies in their fine-grained pipelining and massive parallelism. FPGAs can provide the freedom to fully customize the circuit to fit a specific application. Integer and bit operations are more suitable for a FPGA implementation. However, like any implementation, to achieve the best possible performance, the original algorithm needs to be carefully tailored to fit the specific architecture(s).

## Conclusions

In this paper, we have presented a novel hybrid system to accelerate the mapping of short reads to a reference genome based on the seed-and-extend approach. We propose a parallel banded semi-global alignment architecture to accelerate the computation-intensive extension stage on an FPGA. Meanwhile, we also apply a bucket hash structure to improve the seed generation stage computation. The performance comparison to the GASSST and BWA software implementations under different test conditions shows that our FPGA aligner achieves a high degree of sensitivity and requires less overall execution time with only modest resource utilization. As a result, the performance bottleneck on the FPGA aligner changes from the extension stage to the seed generation stage.

As part of our future work, we are planning to expand the functionality of our FPGA aligner to cope with longer read datasets. Meanwhile, we will also investigate the possibility to integrate the BWT-based search algorithm into our seed generation stage design to reduce the memory consumption.

## Endnote

^a^Normally, there are three types of alignment errors: insertion, deletion, and substitution.

## Competing interests

There is no competing interests for this manuscript.

## Authors’ contributions

YC conceptualized the study, carried out the design and implementation of the architecture, performed the performance tests, analyzed the results and drafted the manuscript; BS and DLM conceptualized the study, participated in the analysis of the results and contributed to the revising of the manuscript. All authors read and approved the final manuscript.
